# Hydrangenol Isolated from the Leaves of *Hydrangea serrata* Attenuates Wrinkle Formation and Repairs Skin Moisture in UVB-Irradiated Hairless Mice

**DOI:** 10.3390/nu11102354

**Published:** 2019-10-02

**Authors:** Da-Bin Myung, Hee-Soo Han, Ji-Sun Shin, Ji Yeon Park, Han Jun Hwang, Hyoung Ja Kim, Hye Shin Ahn, Sun Hee Lee, Kyung-Tae Lee

**Affiliations:** 1 Department of Pharmaceutical Biochemistry, College of Pharmacy, Kyung Hee University, Seoul 02447, Korea; dabin_happy@naver.com (D.-B.M.); heesu3620@hanmail.net (H.-S.H.); jsunvet@naver.com (J.-S.S.); yoolii@daum.net (J.Y.P.); plysolo@naver.com (H.J.H.); 2 Department of Life and Nanopharmaceutical Science, College of Pharmacy, Kyung Hee University, Seoul 02447, Korea; 3 Department of Fundamental Pharmaceutical Sciences, College of Pharmacy, Kyung Hee University, Seoul 02447, Korea; 4 Molecular Recognition Research Center, Materials and Life Science Research Division, Korea Institute of Science and Technology, Seoul 02792, Korea; khj@kist.re.kr; 5 Department of New Material Development, COSMAXBIO, Seongnam 13486, Korea; hsahn@cosmax.com

**Keywords:** hydrangenol, ultraviolet B, photoaging, MMPs, collagen, skin moisture, wrinkle formation, MAPK, AP-1

## Abstract

Our previous study showed that hydrangenol isolated from *Hydrangea serrata* leaves exerts antiphotoaging activity in vitro. In this study, we determined its antiphotoaging effect in UVB-irradiated HR-1 hairless mice. We evaluated wrinkle formation, skin thickness, histological characteristics, and mRNA and protein expression using qRT-PCR and Western blot analysis in dorsal skins. Hydrangenol mitigated wrinkle formation, dorsal thickness, dehydration, and collagen degradation. Hydrangenol increased the expression of involucrin, filaggrin, and aquaporin-3 (AQP3) as well as hyaluronic acid (HA) production via hyaluronidase (HYAL)-1/-2 downregulation. Consistent with the recovery of collagen composition, the expression of Pro-COL1A1 was increased by hydrangenol. Matrix metalloproteinase (MMP)-1/-3, cyclooxygenase-2 (COX-2), and interleukin-6 (IL-6) expression was reduced by hydrangenol. Hydrangenol attenuated the phosphorylation of mitogen-activated protein kinases (MAPKs) including ERK and p38, activator protein 1 (AP-1) subunit, and signal transduction and activation of transcription 1 (STAT1). Hydrangenol upregulated the expression of nuclear factor-E2-related factor 2 (Nrf2), heme oxygenase-1 (HO-1), NAD(P)H quinone dehydrogenase 1 (NQO-1), glutamate cysteine ligase modifier subunit (GCLM), and glutamate cysteine ligase catalysis subunit (GCLC). Taken together, our data suggest that hydrangenol can prevent wrinkle formation by reducing MMP and inflammatory cytokine levels and increasing the expression of moisturizing factors and antioxidant genes.

## 1. Introduction

The human skin protects the body from mechanical damage, penetration of fungi and radiation, and reduces moisture loss, serving as an important barrier to maintaining homeostasis constancy. Skin aging is categorized into extrinsic and intrinsic aging. Intrinsic aging follows a natural course and is determined by internal factors such as genes, hormones, and metabolism. Extrinsic aging (photoaging) is mainly caused by ultraviolet (UV) exposure and is characterized by degradation of skin structure and increase in thickness, sagging, roughness, coarse wrinkle formation, and dehydration [1]. The epidermal barrier to water loss is largely dependent on the stratum corneum (SC), the outermost layer of the epidermis [2]. Involucrin and filaggrin are chiefly associated with skin barrier function. Involucrin is a constituent of the cornified envelope (CE), which is a key component of the SC, and filaggrin makes up the SC and increases the levels of natural moisturizing factors (NMFs), thus strengthening the skin barrier by maintaining moisture [3,4]. Ultraviolet B (UVB) irradiation destroys skin barrier function by affecting involucrin and filaggrin, thereby increasing transepidermal water loss (TEWL), resulting in the disruption of the SC integrity [5,6,7].

Aquaporins (AQPs) are cell membrane proteins that act as selective pores allowing water, glycerol, and other small molecules to pass through the cell membrane. Among the aquaphorins, aquaphorin-3 (AQP3) is the most abundant aquaglyceroporin expressed in the epidermis and plays an important role in skin hydration, as it transports water and glycerol [8]. AQP3 expression is downregulated by UV irradiation in skin keratinocytes and skin fibroblasts, resulting in skin dehydration and a delayed wound-healing response [2,9]. Hyaluronic acid (HA), a bountiful component of the extracellular matrix of the skin, plays an important role in skin hydration and contributes to the structural integrity of the skin [10]. Previous studies have shown that HA degradation occurs due to an increase in the levels of reactive oxygen species (ROS) and HA degrading enzymes, hyaluronidases (HYALs), following UVB irradiation [11]. The dermis is the largest layer of the skin, the bulk of which is a connective tissue matrix primarily made up of collagen, which provides mechanical strength, elasticity, and resiliency to the skin [12]. One of the most abundant proteins in the skin, type I collagen, functions as a membrane for guided tissue regeneration [13]. UV irradiation initiates the fragmentation of collagen, leading to the accumulation of collagen damage, and therefore affects wrinkle formation [14].

ROS generated by UVB is an important pathogenic factor in skin aging. Increased ROS levels lead to oxidative stress, collapse of the extracellular matrix (ECM) components, and initiate signal transduction pathways, such as mitogen-activated protein kinases (MAPKs), signal transduction and activation of transcription (STAT), and the nuclear factor erythroid 2-related factor 2 (Nrf2) signal cascades [15]. Activation of MAPKs results in the phosphorylation and upregulation of the activator protein-1 (AP-1), which promotes the expression of matrix metalloproteinases (MMPs). MMPs belong to the zinc-containing endopeptidases with an extensive range of substrate specificities and play vital roles in skin wrinkle formation by triggering the degradation of ECM. MMP-1 is also called type I collagenase and plays a role in the breakdown of type I and III collagen. MMP-3, also known as stromelysin-1, is responsible for the degradation of type I collagen and the activation of MMP-1, -9, and pro-MMPs [16,17]. UVB also stimulates STATs, resulting in the upregulation of COX-2 and inflammatory cytokines such as IL-6 [18]. Because UVB irradiation increases the generation of ROS and eventually leads to photoaging, mammals stimulate Nrf2/antioxidant response elements (AREs)-mediated endogenous antioxidant systems, which express heme oxygenase-1 (HO-1), NAD(P)H quinone dehydrogenase 1 (NQO-1), glutamate cysteine ligase modifier subunit (GCLM), and glutamate cysteine ligase catalysis subunit (GCLC), to prevent oxidative stress induced by ROS, thereby reducing photoaging or skin damage [19,20]. The Kelch-like enoyl-CoA hydratase (ECH)-associated protein 1 (Keap1) protein regulates the activation of Nrf2 by holding it in the cytoplasm under dormant conditions. Under the oxidative stress caused by an inducer such as UV, Nrf2 detaches from Keap1, translocates to the nucleus, and upregulates the expression of various antioxidant enzymes such as HO-1, NQO-1, GCLM, and GCLC [21].

**Figure 1:**
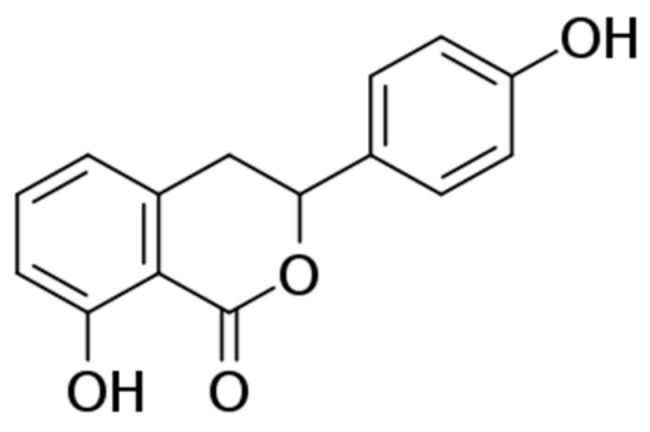
Chemical structure of hydrangenol.

Hydrangenol (Figure 1), a natural dihydroisocoumarin, is mostly obtained from the leaves of hydrangea (Hydrangeaceae). Some researchers have reported that hydrangenol possesses antifungal [22], anti-allergic [23], antidiabetic [24], anti-inflammatory, [25] and anti-angiogenic activities [26]. In our previous study, hydrangenol isolated from the leaves of *Hydrangea serrata* (Thunb.) Ser showed potent antiphotoaging activities in Hs68 human fibroblasts by suppressing MAPK/AP-1 and STAT1 signaling [27]. Therefore, we evaluated the antiphotoaging effect of hydrangenol on wrinkle formation and skin dehydration by evaluating various parameters in UVB-irradiated hairless mice.

## 2. Materials and Methods

### 2.1. Preparation of Hydrangenol from the Leaves of Hydrangea serrata

The dried leaves of *Hydrangea serrata* (Thunb.) Ser. (18 kg) were extracted with distilled water (360 L) at 98 °C for 5 h (WHS, 3.6 kg). WHS (3 kg) was fractionated on a Diaion HP-20 column, starting with 30% MeOH followed by 50% (50 M), 70% (70 M), 100% MeOH (100 M), and CH_2_Cl_2_/MeOH 1:1 as an eluent, giving five fractions (Fr-1–Fr-5). Fraction 4 (200 g) was subjected to a Sephadex LH-20 gel column eluted with MeOH to afford seven subfractions (Fr-4a–Fr-4g). Hydrangenol (15 g, Figure 1) was obtained by recrystallization of Fr-4d (20 g) in MeOH. The qualitative and quantitative analyses were carried out to identify the hydrangenol by comparison of their high performance liquid chromatography (HPLC) retention times and NMR spectroscopic data with authentic reference hydrangenol as shown in Figures S1 and S2. Its purity was determined by HPLC (purity: >97%).

### 2.2. Animal and Treatment

HR-1 hairless mice (male, 5 weeks old) were obtained from SLC Inc. (Shizuoka, Japan). All mice were bred under consistent conditions (temperature: 22 ± 1 °C, humidity: 40%–60%, light/dark cycle: 12 h). All experimental procedures for animal experiments were controlled in accordance with university guidelines of the ethical committee for Animal Care and Use of the Kyung Hee University (KHUASP(SE)-18-107). Hydrangenol was dissolved in a vehicle solution (dimethyl sulfoxide (5%) + kolliphor EL (5%) + sterilized water (90%)) and administered per os (p.o.). After acclimatization for one week, the mice were randomly separated into six groups (*n* = 8): Vehicle-treated control (without UVB irradiation), UVB + vehicle-treated, UVB + Hydrangenol (5 mg/kg/day, p.o.), UVB + Hydrangenol (10 mg/kg/day, p.o.), UVB + Hydrangenol (20 mg/kg/day, p.o.) and UVB + Hydrangenol (40 mg/kg/day, p.o.). 

### 2.3. UVB Irradiation

HR-1 hairless mice were exposed to UVB three times a week for 7 weeks using a UVP Croslinker (Analytik Jena AG, Jena, Germany). The UVB energy was progressively increased from 60 mJ/cm^2^ (1st to 2rd weeks), to 120 mJ/cm^2^ (3rd to 4th weeks), to 180 mJ/cm^2^ (5th to 6th weeks), and to 240 mJ/cm^2^ (7th week).

### 2.4. Evaluation of Skin Wrinkle Formation

Shortly after sacrifice, skin replicas were made from the back skin of hairless mice using SILFLO (Flexico, Colchester, UK) and then analyzed to measure skin roughness. The skin wrinkles were evaluated in accordance with the following indicators; total wrinkle area, mean length, mean depth, and max wrinkle depth.

### 2.5. Histological Analysis

The dorsal sample was taken, fixed overnight with 4% paraformaldehyde, dehydrated in ethanol, and then embedded into the paraffin and sliced thinly. The skin layer changes were analyzed through hematoxylin and eosin (H&E) staining and collagen fibers through Masson’s trichrome staining at the Seoul Medical Science Institute (SCL. Co. Ltd., Seoul, Korea).

### 2.6. Skin Hydration and Skin Transepidermal Water Loss (TEWL) Analysis

The amount of skin moisture and TEWL was determined using a GPSkin Barrier^®^ (GPOWER Inc., Seoul, Republic of Korea) from the back skin of the mouse on the last day of UVB exposure. 

### 2.7. Measurement of Hyaluronic Acid and Procollagen Type I Production

The productions of hyaluronic acid and procollagen type I in skin tissues were measured using (TakaRa, Shiga, Japan) and (R & D Systems, Minneapolis, MN, USA).

### 2.8. Western Blot Analysis

The collected skin tissues were homogenized for protein extraction. Total proteins (25–35 μg) were used for analysis of the expression or phosphorylation of proteins. The protein separation, transfer, and detection process was performed as previously reported [27]. The phospho-c-Fos (#5348), phospho-STAT1 (#8826), phospho-p38 (#9215), phospho-c-Jun (#9261), and HO-1 (#70081) antibodies were purchased from Cell Signaling Technology, Inc. (Danvers, MA, USA) and Pro-COL1A1 (sc-25973), c-Fos (sc-253), c-Jun (sc-74543), STAT1 (sc-592), p38 (sc-271120), phospho-c-Jun N-terminal kinases (p-JNK) (sc-6254), JNK (sc-7345), phospho-extracellular signal-regulated kinases (p-ERK) (sc-7383), ERK (sc-94), Nrf2 (sc-722), and β-actin (sc-81178) antibodies from Santa Cruz Biotechnology (Dallas, Texas, CA, USA). The MMP-1 and MMP-3 protein expression were detected using antibodies from Biolegends^®^ (San Diego, CA, USA). The optical band density of each representative band was calculated in proportion to the internal control or total form of the corresponding protein.

### 2.9. Quantitative Real-Time Polymerase Chain Reaction (PCR) Analysis

The skin tissues of mice were collected, and the tissues were homogenized in Easy Blue^®^ kits (Intron Biotechnology, Seoul, Korea) for RNA extraction. The cDNA was synthesized using 0.5 mg/mL random oligonucleotide primers (Promega, Madison, WI, USA) and TOPscript^TM^ RT DryMIX (Enzynomics, Daejeon, Korea). The expression of the target genes was analyzed by measuring the incorporation of SYBR (SYBR Premix Ex Taq, TakaRa, Shiga, Japan) relative to the internal control β-actin using the Takara thermal cycler device. The PCR primers (Table S1) were designed using the Primer 3 program and custom-made in Bioneer (Daejeon, Republic of Korea)

### 2.10. Statistical Analysis

The values of all experimental results expressed the results of each mouse as the mean ± SD (*n* = 8), and the significance of the difference between each mean value was performed by SigmaPlot software *t*-test analysis. The *p*-value (<0.05) was determined to be statistically significant.

## 3. Results

### 3.1. Hydrangenol Mitigates Wrinkle on the UVB-Irradiated Dorsal Skin of HR-1 Hairless Mice

**Figure 2:**
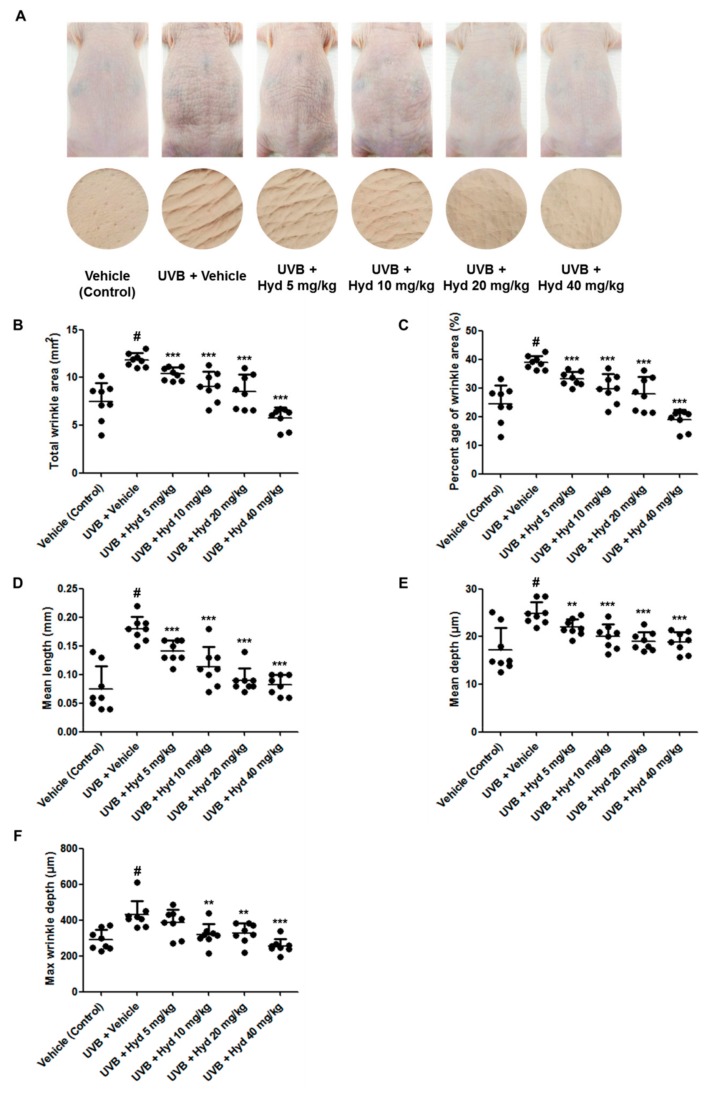
Effects of oral administration of hydrangenol on UVB-induced wrinkles in hairless mice. HR-1 hairless mice were administered hydrangenol (5, 10, 20, or 40 mg/kg) daily for 7 weeks of exposure to ultraviolet B (UVB). (**A**) Representative images of dorsal skin surface and replicas exposed to UVB. (**B**–**F**) The parameters for wrinkle formation, including (**B**,**C**) total wrinkle area, (**D**) mean length, (**E**) mean depth, and (**F**) max wrinkle depth, were obtained from the skin replica analysis. Values are expressed as means ± SD (*n* = 8). # *p* < 0.05 vs. the vehicle-treated control group; ** *p* < 0.01, and *** *p* < 0.001 as compared to the UVB + vehicle-treated group.

Repetitive UVB exposure increases skin thickness, collagen fragmentation, and MMP expression, as well as induces skin wrinkle formation and reduces skin barrier function [16,28]. We evaluated the antiphotoaging effects of orally administered hydrangenol on UVB-irradiated HR-1 hairless mice. HR-1 hairless mice were exposed to repeated and stepwise increased UVB irradiation for 7 weeks, which resulted in the wrinkle formation necessary to perform the experiment. Oral administration of hydrangenol (5, 10, 20, or 40 mg/kg) to UVB-irradiated hairless mice reduced wrinkle formation (Figure 2A). After sacrifice, skin replicas were prepared from the dorsal skin layer of the HR-1 hairless mice and the severity of skin wrinkles was quantified by assessing wrinkle parameters including total wrinkle area, percentage of wrinkle area, mean length/depth of the wrinkle, and the maximum wrinkle depth. We found that following 7 weeks of oral hydrangenol (5, 10, 20, or 40 mg/kg) administration, these UVB-induced wrinkle parameters were reduced in a dose-dependent manner (Figure 2B–F).

### 3.2. Hydrangenol Reduces UVB-Induced Epidermis/Dermis Thickness, TEWL, and Skin Dehydration on the Dorsal Skin of HR-1 Hairless Mice

**Figure 3:**
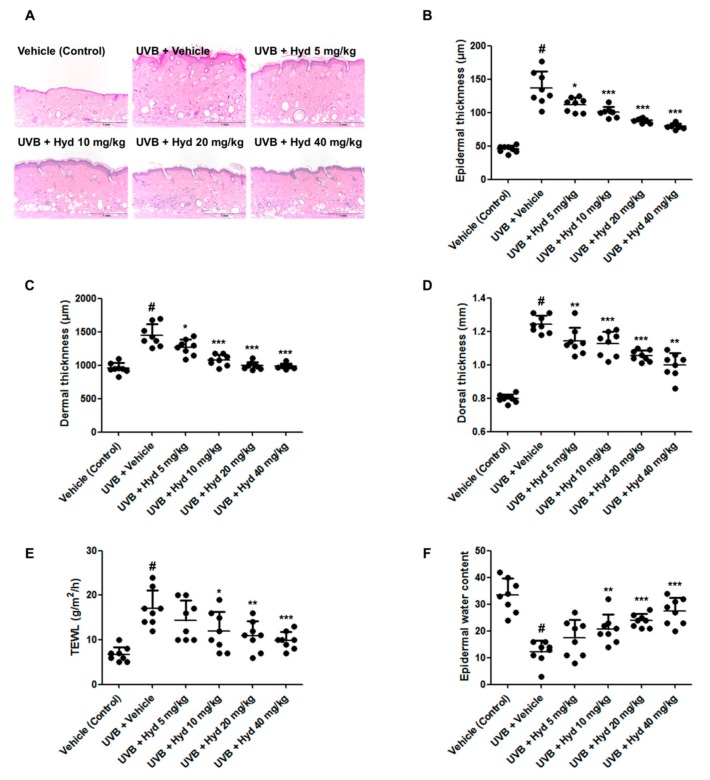
Effects of oral administration of hydrangenol on skin thickness, skin hydration, and transepidermal water loss (TEWL) in UVB-irradiated hairless mice. HR-1 hairless mice were administered hydrangenol (5, 10, 20, or 40 mg/kg) daily and exposed to UVB irradiation three times a week for 7 weeks. Skin tissues were stained with (**A**) hematoxylin and eosin (H&E) to aid observation of (**B**) epidermal thickness and (**C**) dermal thickness of each group of mice. (**D**) The dorsal thickness of hairless mice was measured using a caliper before sacrifice. (**E**) TEWL and (**F**) epidermal water content were measured on the dorsal surface area of the mice. Values are expressed as means ± SD (*n* = 8). # *p* < 0.05 vs. the vehicle-treated control group; * *p* < 0.05, ** *p* < 0.01, and *** *p* < 0.001 as compared to the UVB + vehicle-treated group.

To investigate the suppressive effects of hydrangenol on skin thickness, a dorsal skin section of HR-1 hairless mice was stained with hematoxylin and eosin (H&E). H&E staining revealed that epidermal and dermal thickness in the UVB-irradiated group was evidently higher than that in the vehicle-treated control group. Interestingly, the hydrangenol-administered (5, 10, 20, or 40 mg/kg) group demonstrated a significant reduction in the epidermal, dermal, and total skin thickness (Figure 3A–D). We further examined the effects of hydrangenol on epidermal barrier function by measuring TEWL and epidermal hydration. UVB irradiation for 7 weeks increased TEWL but decreased epidermal water content. However, oral administration of hydrangenol (10, 20, or 40 mg/kg) significantly reduced the UVB-induced TEWL and improved epidermal water content (Figure 3E,F).

### 3.3. Hydrangenol Increases Skin Barrier Gene Expression and HA Production Via Downregulating HYAL mRNA Expression in UVB-Irradiated HR-1 Hairless Mice

**Figure 4:**
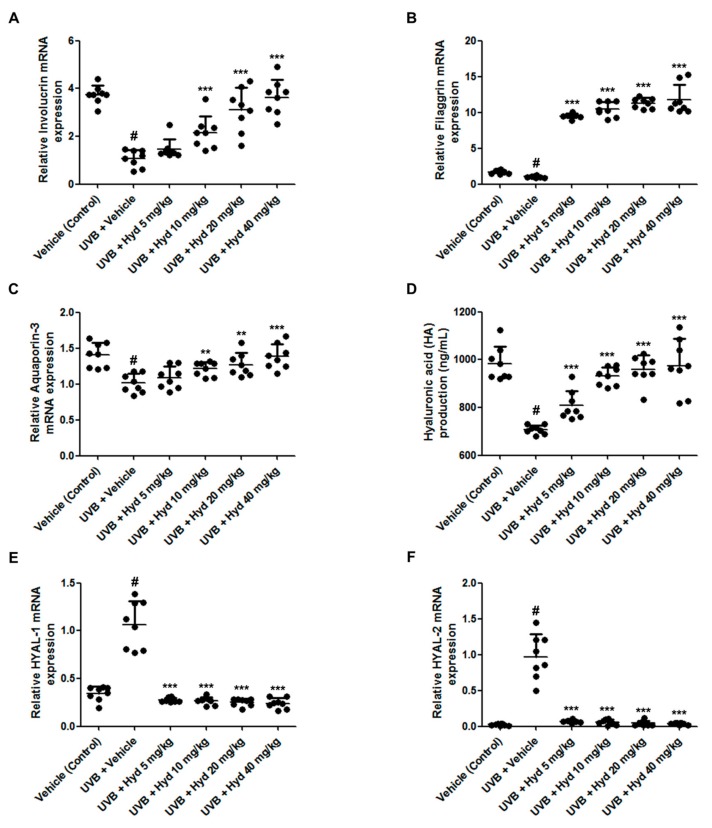
Effects of oral administration of hydrangenol on hyaluronic acid content, involucrin, filaggrin, aquaporin-3 (AQP3), and hyaluronidase (HYAL)-1/-2 mRNA expression in UVB-irradiated hairless mice. HR-1 hairless mice were administered hydrangenol (5, 10, 20, or 40 mg/kg) daily for 7 weeks of exposure to UVB. Total proteins and RNA were extracted from dorsal skin tissues. The mRNA levels of (**A**) involucrin, (**B**) filaggrin, (**C**) AQP3, (**E**) HYAL-1, and (**F**) HYAL-2 were analyzed by qRT-PCR. (**D**) The protein lysates were used to measure hyaluronic acid production. Values are expressed as means ± SD (*n* = 8). # *p* < 0.05 vs. the vehicle-treated control group; ** *p* < 0.01, and *** *p* < 0.001 as compared to the UVB + vehicle-treated group.

Loss of skin moisture following UVB exposure is closely related to dysregulation in the expression of skin barrier proteins in the SC. Therefore, we evaluated the effects of hydrangenol on the expression of involucrin, filaggrin, and AQP3, which are major constituents of the epidermal barrier and are responsible for skin hydration [2,29], in UVB-irradiated HR-1 hairless mice. As expected, UVB irradiation decreased the mRNA expression levels of involucrin, filaggrin, and AQP3; however, oral administration of hydrangenol increased their expression in a dose-dependent manner (Figure 4A–C). Because HA, a key component of the dermal ECM, controls skin functions such as skin hydration and elasticity, and is therefore associated with UVB-induced skin aging [10], we further examined the effects of hydrangenol on UVB-induced reduction of HA production. We observed that oral administration of hydrangenol (5, 10, 20, or 40 mg/kg) significantly increased HA production in UVB-irradiated HR-1 hairless mice (Figure 4D). Next, we examined the effects of hydrangenol on the mRNA levels of HYAL in the dorsal skin of UVB-induced HR-1 hairless mice. As shown in Figure 4E,F, the mRNA levels of HYAL1 and HYAL2 were increased following UVB irradiation, but the oral administration of hydrangenol reduced their mRNA levels, suggesting that hydrangenol treatment recovered the loss of HA via reduction of UVB-induced HA degradation. These results indicate that hydrangenol significantly inhibits UVB-induced skin dehydration by upregulating the expression of involucrin, filaggrin, and AQP3 as well as HA production.

### 3.4. Hydrangenol Restores Pro-Collagen Type I Production and Pro-COL1A1 Protein and mRNA Expression in UVB-Irradiated HR-1 Hairless Mice

**Figure 5:**
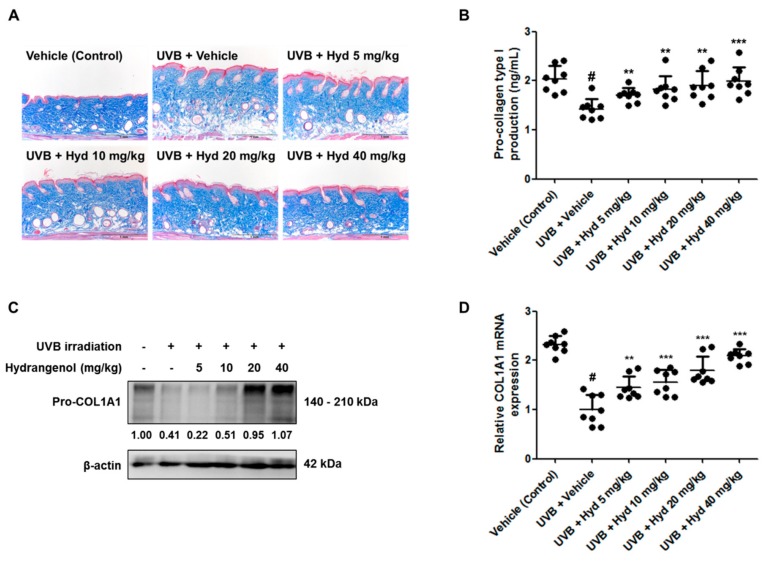
Effects of oral administration of hydrangenol on collagen content in UVB-irradiated hairless mice. HR-1 hairless mice were administered hydrangenol (5, 10, 20, or 40 mg/kg) daily for 7 weeks of exposure to UVB. (**A**) Skin tissues were subjected to Masson’s trichrome staining to observe collagen fibers of each group of mice. Total proteins and RNA were extracted from dorsal skin tissues. The protein lysates were used to measure (**B**) procollagen type 1 production. (**C**) The protein expression of Pro-COL1A1 was estimated by Western blotting using a specific Pro-COL1A1 antibody. The internal control was a β-actin protein. Each band is representative of three experiments. (**D**) COL1A1 mRNA levels were analyzed by qRT-PCR. Values are expressed as means ± SD (*n* = 8). # *p* < 0.05 vs. the vehicle-treated control group; ** *p* < 0.01, and *** *p* < 0.001 as compared to the UVB + vehicle-treated group.

With repetitive UVB irradiation, dermal collagen is degraded and consequently, the spatial density of collagen is reduced. To estimate the effects of hydrangenol on UVB-induced collagen degradation, the dorsal skin sections of the HR-1 hairless mice were stained with Masson’s trichrome. We observed that UVB-induced reduction in collagen fibers was restored after oral administration of hydrangenol (Figure 5A). As shown in Figure 5B, the oral administration of hydrangenol prevented UVB-induced reduction in pro-collagen type I production in a dose-dependent manner. To further elucidate the recovery mechanism of hydrangenol on pro-collagen type I, we measured the protein and mRNA expression of COL1A1, an important component in collagen formation [30], by Western blotting and qRT-PCR, respectively. Hydrangenol increased both the protein and mRNA levels of Pro-COL1A1 in UVB-irradiated hairless mice (Figure 5C,D). These data demonstrate that hydrangenol promoted the production of pro-collagen type I by upregulating COL1A1 gene expression.

### 3.5. Hydrangenol Attenuates UVB-Induced Expression of MMP-1/-3, COX-2, and IL-6 in HR-1 Hairless Mice

**Figure 6:**
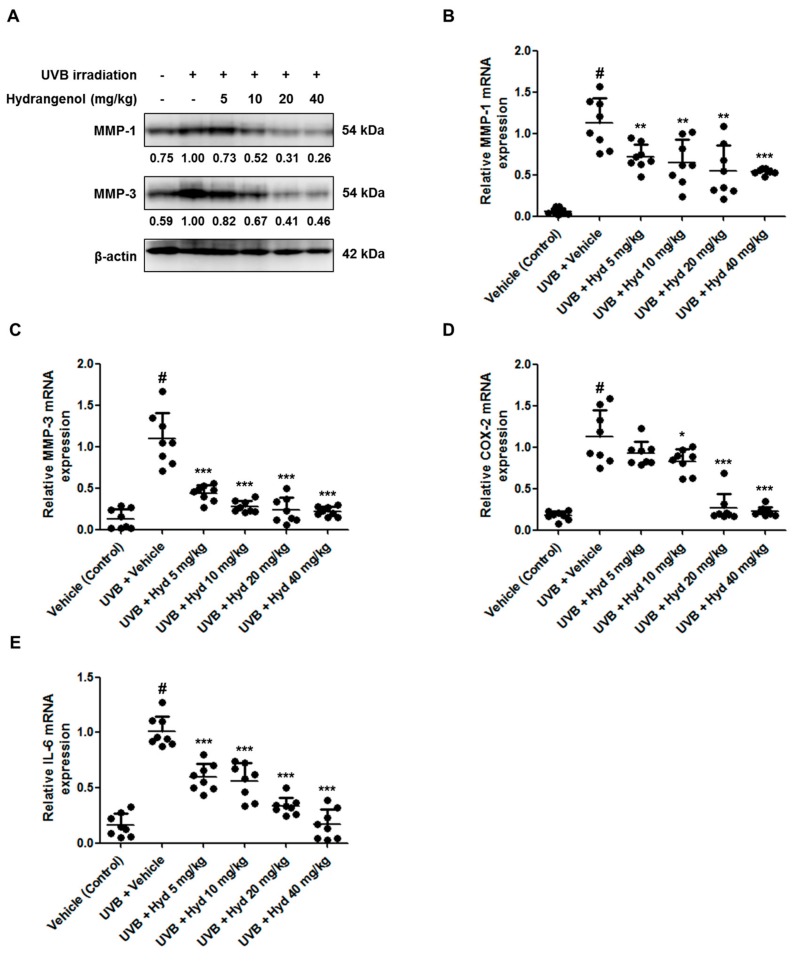
Effects of oral administration of hydrangenol on matrix metalloproteinase (MMP)-1/-3, COX-2, and IL-6 expression in UVB-irradiated hairless mice. HR-1 hairless mice were administered hydrangenol (5, 10, 20, or 40 mg/kg) daily for 7 weeks of exposure to UVB. Total proteins and RNA were extracted from dorsal skin tissues. (**A**) The protein expression of MMP-1 and MMP-3 were estimated by Western blotting using specific MMP-1 and MMP-3 antibodies. The internal control was a β-actin protein. Each band is representative of three experiments. (**B**,**C**) The mRNA levels of MMP-1/-3, (**D**,**E**) COX-2, and IL-6 were analyzed by qRT-PCR. Values are expressed as means ± SD (*n* = 8). # *p* < 0.05 vs. the vehicle-treated control group; * *p* < 0.05, ** *p* < 0.01, and *** *p* < 0.001 as compared to the UVB + vehicle-treated group.

MMP-1 and MMP-3 are protease enzymes that are capable of degrading native fibrillar collagens in the human skin [17]. Here, we examined whether hydrangenol inhibits the expression these MMPs at the protein and mRNA levels in UVB-irradiated HR-1 hairless mice using Western blotting and qRT-PCR, respectively. As shown in Figure 6A–C, oral administration of hydrangenol significantly reduced UVB-induced MMP-1/-3 protein and mRNA levels. In addition, UVB exposure upregulated the expression levels of pro-inflammatory mediators (COX-2 and ILs), which cause skin damage [18]. To elucidate the effects of hydrangenol on skin inflammation, we measured COX-2 and IL-6 mRNA levels by qRT-PCR. In the hydrangenol-administered group, UVB-induced mRNA expression of COX-2 and IL-6 was suppressed in a dose-dependent manner (Figure 6D,E).

### 3.6. Hydrangenol Prevents the Activation of STAT1 and AP-1 and the MAPK Signaling Pathway in UVB-Induced HR-1 Hairless Mice

**Figure 7:**
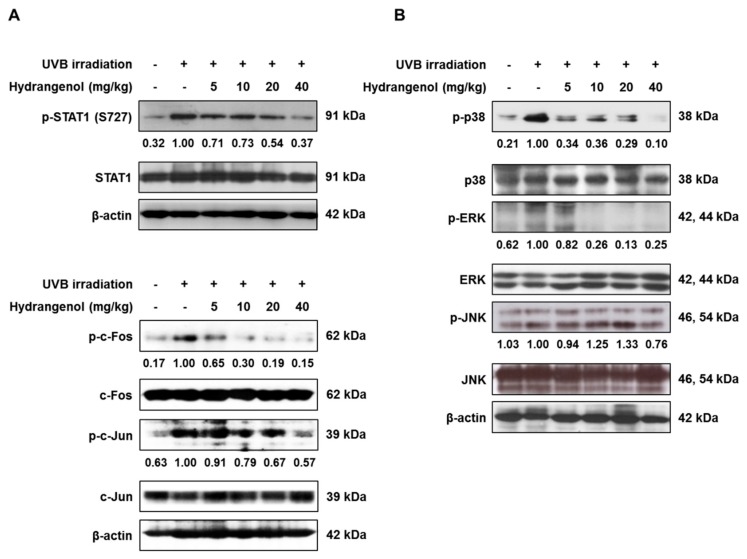
Effects of oral administration of hydrangenol on STAT1, AP-1, and the mitogen-activated protein kinase (MAPK) signaling pathway in UVB-irradiated hairless mice. HR-1 hairless mice were administered hydrangenol (5, 10, 20, or 40 mg/kg) daily for 7 weeks of exposure to UVB. Total proteins were extracted from dorsal skin tissues. The phosphorylation and expression of target proteins were estimated using Western blotting using specific p-STAT1 (S727), p-c-Fos, p-c-Jun, p-p38, phospho-extracellular signal-regulated kinase (p-ERK), and phospho-c-Jun N-terminal kinase (p-JNK) antibodies. The internal control was a β-actin protein. Representative blots of (**A**) STAT1, AP-1, and (**B**) MAPK signaling proteins. Each band is representative of three experiments.

As it is known that the activation of STAT1 and AP-1 promotes UVB-induced skin inflammation and the transcription of MMP-1 and MMP-3 [31], we examined the effects of hydrangenol on the phosphorylation of STAT1 and AP-1 proteins (Fos–Jun heterodimer) to determine the molecular pathways involved in the antiphotoaging mechanisms of hydrangenol in UVB-irradiated HR-1 hairless mice. Our results showed that hydrangenol inhibited the phosphorylation of STAT1 and c-Fos and c-Jun, subunits of AP-1 (Figure 7A). We further demonstrated that hydrangenol inhibited the phosphorylation of p38 and ERK, but not of JNK. However, the expressions of the total p38, JNK, and ERK1/2 were unaffected by UVB irradiation with or without hydrangenol (Figure 7B). Our results elucidated that hydrangenol might inhibit MMP expression by downregulating the activation of p38, ERK, AP-1, and STAT1 signaling in UVB-irradiated HR-1 hairless mice.

### 3.7. Hydrangenol Promotes the Nrf2/ARE Signaling Pathway in UVB-Induced HR-1 Hairless Mice

**Figure 8:**
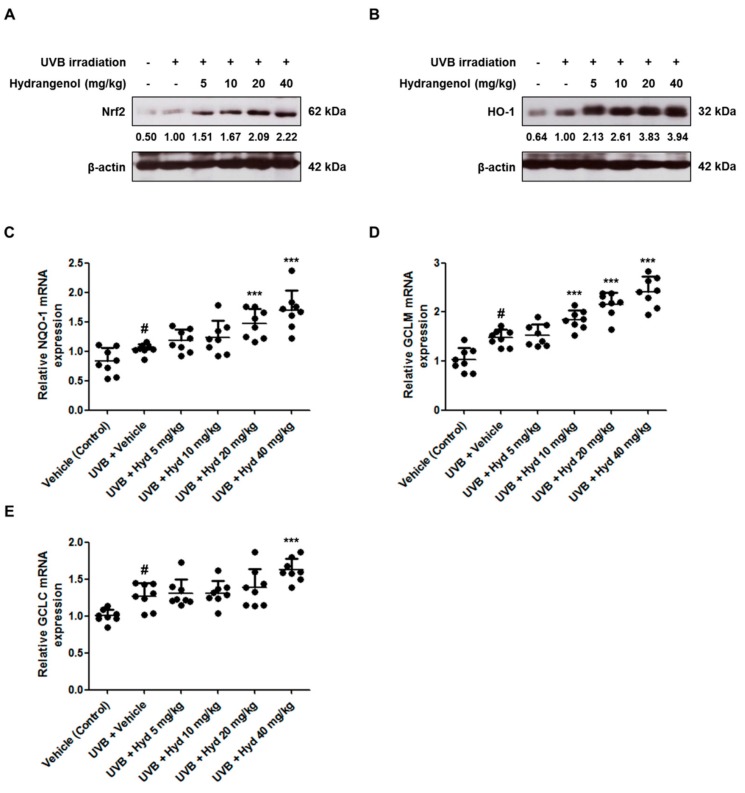
Effects of oral administration of hydrangenol on the Nrf2/ARE signaling pathway in UVB-irradiated hairless mice. HR-1 hairless mice were administered hydrangenol (5, 10, 20, or 40 mg/kg) daily for 7 weeks of exposure to UVB. Total proteins were extracted from dorsal skin tissues. (**A**,**B**) The expression of target proteins was estimated using Western blotting using specific Nrf2 and HO-1 antibodies. The internal control was a β-actin protein. Representative blots of (**A**) Nrf2 and (**B**) HO-1 expression. Each band is representative of three experiments. (**C**–**E**) The mRNA levels of NQO-1, GCLM, and GCLC were analyzed by qRT-PCR. Values are expressed as means ± SD (*n* = 8). # *p* < 0.05 vs. the vehicle-treated control group; *** *p* < 0.001 as compared to the UVB + vehicle-treated group.

Nrf2 has been suggested to play a protective role in UV-mediated oxidative stress [32]. Under oxidative stress, the transcription factor Nrf2 promotes the gene expression of phase II enzymes that resist oxidative stress, such as HO-1, NQO-1, GCLM, and GCLC [33,34]. To examine whether the photoprotective effects of hydrangenol were mediated by Nrf2 activation, we evaluated the expression of Nrf2 and Nrf2-regulated antioxidant genes in the hydrangenol-administered groups. The protein expression of Nrf2 in the UVB-irradiated group was slightly increased compared to that in the vehicle-treated control group. Hydrangenol administration (5, 10, 20, or 40 mg/kg) increased Nrf2 protein expression in a dose-dependent manner compared with the only UVB-irradiated group (Figure 8A). With Nrf2 activation, HO-1 protein levels and NQO-1, GCLM, and GCLC mRNA levels were slightly upregulated following UVB irradiation. These levels were further upregulated with hydrangenol treatment (Figure 8B–E).

## 4. Discussion

As the aging population is expanding worldwide, there is increased interest in anti-aging agents and photoprotective oral dietary supplements based on plant extracts and natural compounds, so as to minimize and prevent the manifestations of aging [35]. The skin is constantly affected by solar UV irradiation and various environmental stresses, which in turn promote skin aging [12]. In this study, we found that oral administration of hydrangenol for 7 weeks reduced wrinkle parameters including total wrinkle area, percentage of wrinkle area, mean length/depth of the wrinkle, and maximum wrinkle depth, and restored skin thickness and dehydration in the dorsal skin of UVB-irradiated hairless mice, consequently providing protection against photoaging. However, the current experiment did not confirm the infiltration of inflammatory cells or changes in cell composition in UVB-irradiated hairless mice.

High moisture retention is an important factor in improving skin barriers. As hydrangenol administration significantly increased skin hydration, we investigated the skin barrier-related markers including involucrin, filaggrin, and AQP3. Upregulation of these factors might reduce skin aging and wrinkling, possibly by increasing moisture content and improving the skin barrier [36,37]. We observed that treatment with hydrangenol upregulated UVB-reduced mRNA expression of involucrin, filaggrin, and AQP3. Interestingly, the increased expression of filaggrin was considerably higher than that of involucrin and AQP3. Previous reports indicated a significant role of GATA3 in the formation and maintenance of a complete epidermal barrier [38,39,40]. In addition, it was reported that the upregulation of GATA3 significantly enhanced the expression of filaggrin in human keratinocytes [41]. We investigated whether hydrangenol increased the expression of GATA3, but did not find any effect of hydrangenol on the expression of GATA3. This suggests that hydrangenol does not regulate filaggrin through GATA3, but may be controlled by other factors. Our study indicates that hydrangenol can be expected to improve skin barrier function and moisturizing by increasing involucrin, filaggrin, and AQP3 content in UVB-irradiated skin.

An ECM molecule related to skin moisture, HA, plays an essential role in maintaining the hydration balance because of its water retention characteristics, and therefore skin hydration critically depends on the HA-bound water in the dermis [10]. The additional function of HA is to protect skin cells from damage caused by free radicals that greatly affect skin aging [42]. We found that hydrangenol administration effectively recovered the reduction of HA by UVB irradiation in HR-1 hairless mice. Three HA synthases (HAS-1, -2, and -3) are the specific enzymes involved in the synthesis of HA in the internal plasma membranes [43]. HYAL-1 and -2, are the acid-activated enzymes present in plasma membranes and degrades HA into various fragments of varying sizes [44]. In our previous study, hydrangenol demonstrated a potent inductive activity on HA production by decreasing the expression of HYAL-1/-2 [27]. In line with these results, the hydrangenol-administered group reduced the mRNA expression of HYAL-1 and -2 in UVB-irradiated HR-1 hairless mice. The observed increase of HA production by hydrangenol is probably due to the downregulation of HYAL mRNA expression, as no effects on HA synthases (HAS) mRNA expression was observed by hydrangenol compared to the only UVB-irradiated group.

MMP-1 is considered a crucial collagenolytic enzyme which cleaves fibrillary type I collagen [45]. MMP-3 is maximally promoting the activation of latent MMP-1 and subsequently degrades fibrillary type I collagen in the skin [46]. UVB irradiation increases the expression of MMPs, changing the physical properties of skin, including the loss of type I collagen, in the ECM of the dermis [47]. This pattern of change causes skin aging because type I collagen is a major structural protein in the ECM of the dermis and is related to skin elasticity and moisture. In our study, we reported that UVB exposure induced the decrease of procollagen type I and the increase of MMP-1/-3 expression, while this pattern was reversed in the group receiving treatment with hydrangenol. These results suggested that hydrangenol restores collagen levels by decreasing the expression of MMPs and increasing the expression of COL1A1 gene, which regulates type I collagen production, and by increasing the type I collagen precursor, procollagen type I.

Oxidative stress due to UVB irradiation plays a principal role in initiating and driving complex signaling cascades events. The increased levels of ROS induced by UVB irradiation activate various transcriptional factors, including MAPKs, AP-1, STATs, and NF- κB [15,48]. The subunits of AP-1, c-Fos, and c-Jun are activated by a variety of stimuli including growth factors, cytokines, and UV irradiation. Multiple lines of evidence have demonstrated that the transcription of c-Fos and c-Jun depends largely on the activation of the MAPK signaling pathways, which are involved in causing skin aging by strongly regulating the transcription of the several MMPs that degrade collagen [49,50]. In accordance with our previous results in UVB-irradiated Hs68 cells [27], hydrangenol inhibited phosphorylation of p38 and ERK, as well as phosphorylation of c-Fos and c-Jun, AP-1 subunits. Therefore, hydrangenol might inhibit UVB-induced MMP expression by the downregulation of MAPK (p38 and ERK) and AP-1 signaling pathway. In addition, the treatment of the MAPK inhibitors, SB203580 (p38 inhibitor) and PD98059 (ERK inhibitor), suppressed the expression of involucrin, filaggrin, and AQP3 in keratinocytes [2,51,52], implying that their expression is under the control of the MAPK signaling pathway. Therefore, we hypothesize that the inactivation of p38 and ERK by hydrangenol might be involved in restoring the expression of involucrin, filaggrin, and AQP3 as well as MMPs in UVB-irradiated skin.

A previous publication has reported that Nrf2 regulates numerous skin barrier structural and functional components and consequently plays an important role in skin barrier function [32]. It was reported that the activation of Nrf2 is a promising target of photoprotective supplements including bixin [32], tanshinone I [53], *Lycium barbarum* polysaccharide [54], *Periostracum cicadae* [55], zerumbone [56], and gremlin [57]. Since hydrangenol was described in a previous study as reducing ROS in UVB-irradiated Hs68 cells [27] and inhibiting pro-inflammatory mediators by activating Nrf2-mediated HO-1 signaling in lipopolysaccharide-induced BV2 microglial cells [25], to validate this phenomenon in vivo, we studied the inductive effects of hydrangenol on Nrf2 and the expression of Nrf2/ARE-dependent genes in the UVB-irradiated HR-1 hairless mice. Our results demonstrate that hydrangenol promoted the protein expression of Nrf2 and HO-1 in UVB-induced photoaging mice. We identified NQO-1, GCLM, and GCLC as Nrf2-regulated target genes. Interestingly, the inductive effects of hydrangenol on NQO-1, GCLM, and GCLC was weak compared to the effect on HO-1. However, further research will be needed to establish the exact role of hydrangenol-induced Nrf2 and HO-1 using Knockout mice or inhibitors because at present our experiments have not directly addressed the Nrf2/ARE signaling pathway. Given the antiphotoaging effects of hydrangenol and the mechanism of action demonstrated in the animal model, hydrangenol could be a potent antiwrinkle and skin moisturizing agent. In order to accurately describe the efficacy of orally administered hydrangenol, it will be necessary to ensure that sufficient amounts of hydrangenol are attained in skin tissues through the absorption process. Furthermore, further research on the bioavailability and pharmacokinetics of hydrangenol will be needed.

## 5. Conclusions

This study was the first to demonstrate that oral administration of hydrangenol, an active compound of *H. serrata* extracts, effectively prevents wrinkle formation and skin dehydration in UVB-induced photoaging mice. Hydrangenol significantly enhanced the expression of skin hydration factors such as involucrin, filaggrin, AQP3, and HA. Hydrangenol ameliorated UVB-induced collagen deposition by inhibiting the levels of MMPs and inflammatory cytokines via the MAPK/AP-1 and STAT1 signaling pathways. Moreover, hydrangenol reinforced the Nrf2/ARE signal-mediated protection system against oxidative stress. These results support the hypotheses that hydrangenol is a potential therapeutic candidate for improving the conditions of photo-aged skin.

## Supplementary Materials

The following are available online at https://www.mdpi.com/2072-6643/11/10/2354/s1, Table S1: The primer sequences for qRT-PCR, Figure S1: (A) HPLC profile of hydrangenol isolated from WHS and (B) authentic reference hydrangenol, Figure S2: (A) 1H and (B) 13C NMR spectra of hydrangenol isolated from WHS measured in DMSO-d6-H2O.
