# Supplementary material for: Hydrangenol Isolated from the Leaves of Hydrangea serrata Attenuates Wrinkle Formation and Repairs Skin Moisture in UVB-Irradiated Hairless Mice
Source: Nutrients. 2019 Oct 2;11(10):2354. doi: 10.3390/nu11102354 (PMC6835603; doi:10.3390/nu11102354)
Supplement: Supplementary file 1 [file nutrients-11-02354-s001.pdf]

Table S1. The primer sequences for qRT-PCR

Gene	Forward sequence	Reverse sequence
**HYAL1**	5' TACACAGCATGCTCAGAAAG 3'	5' AGTGTCTCCATTCCAAACAG 3'
**HYAL2**	5' AATCTGTGGAACGCTACATC 3'	5' GCCTGCTTCATTACTCTGTC 3'
**Involucrin**	5' GGGTCAGTCACTTAAGCAAG 3'	5' CTACTTCTCCTGCTGTGTCC 3'
**Filaggrin**	5' CTCAGGAGGAAGAGGACAGT 3'	5' CAAGGTGCTTTGCTGTAAAT 3'
**COL1A1**	5' CCCAGAACATCACCTATCAC 3'	5' GAGGTCTTGGTGGTTTTGTA 3'
**MMP-1**	5' TTGCCCAGAGAAAAGCTTCAG 3'	5' TAGCAGCCCAGAGAAGCAACA 3'
**MMP-3**	5' GTTCTGGGCTATACGAGGGC 3'	5' GGCAGCATCGATCTTCTTCA 3'
**COX-2**	5' TGCTGTACAAGCAGTGGCAA 3'	5' GCAGCCATTTCCTTCTCTCC 3'
**IL-6**	5' GAGGATACCACTCCCAACAGACC 3'	5' AAGTGCATCATCGTTGTTCATACA 3'
**NQO-1**	5' TGGCCGATTCAGAGTGGCAT 3'	5' AAACAGGCTGCTTGGAGCAAAA 3'
**GCLM**	5' GGGAACCTGCTCAACTGGGG 3'	5' CTGCATGGGCATGGTGCATT 3'
**GCLC**	5' TCCGGCATCGGAGAGGAGA 3'	5' AGCAGTTGCCCATCCCGAAT 3'
**β-actin**	5' ATCACTATTGGCAACGAGCG 3'	5' TCAGCAATGCCTGGGTACAT 3'

Figure S1.

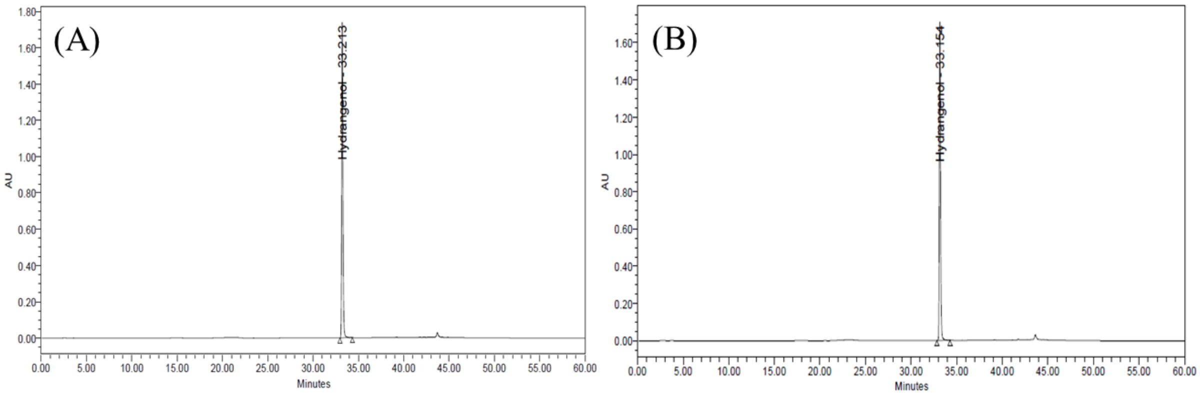

<HPLC analysis conditions>

- Column : Luna C18 (5 µm, 250 × 4.6 mm, Phenomenex)
- Detector : Waters 2489 UV/Vis Detector (228 nm)
- Pump : Waters e2695
- Oven temperature : 30°C
- Injection volume : 20 µL
- Flow rate : 1 mL/min
- Solvent system : Gradient system (linear gradient system)

Time (min)	% A (ACN)	% B (H_2O)
**Initial**	20	80
**15**	25	75
**30**	50	50
**40**	100	0
**50**	20	80

Figure S1. (A) HPLC profile of hydrangenol isolated from WHS and (B) authentic reference hydrangenol

Figure S2.

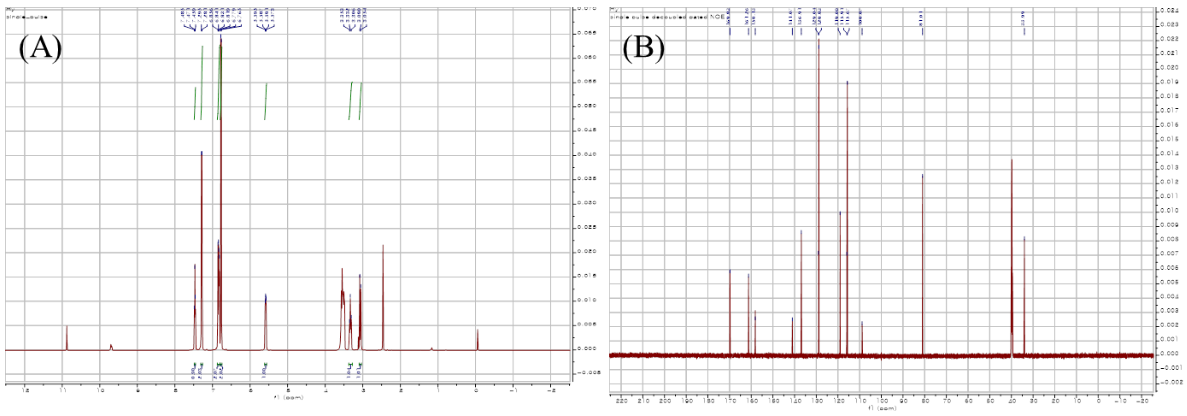

^{1}H-NMR (600 MHz, DMSO-d_6 – D_2O)
δ: 7.47 (1H, dd, *J* = 7.2, 8.4 Hz, H-6), 7.29 (2H, d, *J* = 8.4 Hz, H-2′,6′), 6.85 (1H, d, *J* = 8.4 Hz, H-7), 6.83 (1H, d, *J* = 7.2 Hz, H-5), 6.77 (2H, d, *J* = 8.4 Hz, H-3′,5′), 5.50 (1H, dd, *J* = 3.6, 12.0 Hz, H-3), 3.33 (1H, dd, *J* = 12.0, 15.6 Hz, H-4a), 3.07 (1H, dd, *J* = 3.6, 15.6 Hz, H-4b).

^{13}C-NMR (125 MHz, DMSO-d_6 – D_2O)
δ: 34.0 (C-4), 81.0 (C-3), 108.9 (C-9), 115.7 (C-3′,5′), 115.9 (C-7), 119.0 (C-5), 128.8 (C-2′,6′), 129.0 (C-1′), 136.9 (C-6), 141.1 (C-10), 158.1 (C-4′), 161.3 (C-8), 169.8 (C-1).

Figure S2. (A) 1H and (B) 13C NMR spectra of hydrangenol isolated from WHS measured in DMSO-d6-H2O. The chemical structure of hydrangenol was elucidated by instrumental analyses. 1D-NMR experiments were conducted using (A) 1H-(600 MHz) and (B) 13C-NMR (150 MHz), and recorded with VNS (JEOL, Tokyo, Japan).
